# Impact of growth hormone treatment on a 12-year-old female with newly diagnosed panhypopituitarism and distal arthrogryposis

**DOI:** 10.1530/EDM-25-0146

**Published:** 2026-01-07

**Authors:** Leo L T Meller, Ghassan Akkad, Mary Patterson

**Affiliations:** UC San Diego Health, Rady Children’s Hospital, San Diego, California, USA

**Keywords:** panhypopituitarism, pituitary, growth hormone therapy, distal arthrogryposis, pediatric endocrinology

## Abstract

**Summary:**

Panhypopituitarism, characterized by multiple pituitary hormone deficiencies, is most often diagnosed in infancy or early childhood with adolescent presentation being uncommon. We present a 12-year-old female with late-onset panhypopituitarism presenting with short stature and concomitant bilateral distal arthrogryposis. Her height at presentation was 140.7 cm (6th percentile) with a growth velocity of 0.75 cm/year, Tanner stage 1, bone age consistent with chronological age, and predicted adult height of approximately 152.4 cm despite a mid-parental target near 167.6 cm. Laboratory testing supported growth hormone (GH) deficiency, central hypothyroidism, and adrenal insufficiency, with stimulation tests being subnormal. MRI showed hypoplasia of the anterior pituitary, an absent infundibulum, and an ectopic posterior pituitary, consistent with pituitary stalk interruption syndrome. Hydrocortisone (9 mg/m^2^/day) and levothyroxine (37.5 μg daily) were first initiated. Although GH was initially deferred due to concerns about worsening preexisting distal hand contractures, the family elected to begin weekly subcutaneous lonapegsomatropin-tcgd (Skytrofa, 7.6 mg, 0.24 mg/kg/week). At 3 months, the growth velocity increased to 8.7 cm/year with early breast development, and at 6 months, it reached 17.4 cm/year, bone age remained concordant with chronological age, and predicted adult height improved to approximately 162.6 cm. By 10 months, height percentile rose to the 12th percentile and Tanner stage 2 breast development was observed. Throughout treatment, there were no reported or observed changes in distal arthrogryposis hand contractures. This case report highlights that initiation of GH therapy may lead to a significant growth improvement without aggravating arthrogryposis-related contractures.

**Learning points:**

- To report the rare co-occurrence of arthrogryposis and panhypopituitarism and discuss management.
- Distal arthrogryposis coexisting with GH deficiency did not worsen with GH therapy in this patient, supporting individualized risk–benefit analysis.
- Shared decision making is key when theoretical risks (e.g. joint symptoms) are weighed against the consequences of untreated GH deficiency.

## Background

Panhypopituitarism, characterized by multiple pituitary hormone deficiencies often including growth hormone (GH), thyroid-stimulating hormone (TSH), and adrenocorticotropic hormone (ACTH), has an estimated prevalence between 1 in 16,000 and 1 in 26,000 individuals (1). Most diagnoses occur in infancy or early childhood when delayed developmental milestones first become apparent and prompt evaluation, with a median age of onset varying from the first 3–6 years of life depending on etiology (2, 3). Early diagnosis is crucial, as untreated panhypopituitarism can lead to significant physical and cognitive impairments. The condition may be congenital, due to genetic mutations or structural abnormalities of the pituitary gland, or acquired, stemming from trauma, tumors, or other factors affecting the pituitary (4).

Herein, we present a unique case of a 12-year-old female with late-onset panhypopituitarism presenting with short stature and concomitant bilateral distal arthrogryposis, who was successfully treated with GH without worsening of arthrogryposis contractures. Currently, no reports exist that examine the effects of GH therapy on arthrogryposis contractures. However, clinicians may remain hesitant given concerns about GH-mediated skeletal growth and potential worsening of arthropathies (4). We thus aim to highlight this rare co-occurrence of arthrogryposis and panhypopituitarism and discuss the challenges in managing GH deficiencies in the setting of arthrogryposis.

## Case presentation

A 12-year-old female, born at 38 weeks of gestation with a past medical history of arthrogryposis, presented for an initial endocrinology visit in March 2023 due to concerns about short stature. The patient’s primary care physician initiated the workup after noticing that her growth had significantly lagged behind her mid-parental height expectations. Both parents are 175.3 cm tall, projecting the patient to be at the 75th percentile on the growth curve. Despite this, the patient had been in the 10th–25th percentile for most of her childhood but had recently dropped below the 5th percentile by age 12. She has never experienced a growth spurt disproportionate to her weight gain and overall exhibited a slow, linear growth pattern. In addition, she had not yet reached menarche or started puberty. Despite being a picky eater, she was described as generally healthy, sleeping an average of ten hours a night, and participating actively in physical education classes five times a week. At the time of the initial endocrinology visit, the patient measured 140.7 cm in height (6th percentile), which is 1.57 standard deviations below the mean for her age, with a growth velocity of 0.75 cm/year. Her weight was 30.8 kg (4th percentile) with a BMI of 15.71 kg/m^2^ (13th percentile). On physical examination, she was found to have Tanner stage 1 breast development and Tanner stage 1 pubic hair. Contractures were noted in both hands, in line with her history of arthrogryposis, for which she had been inconsistently following physical and occupational therapy.

## Investigation

**Figure 1:**
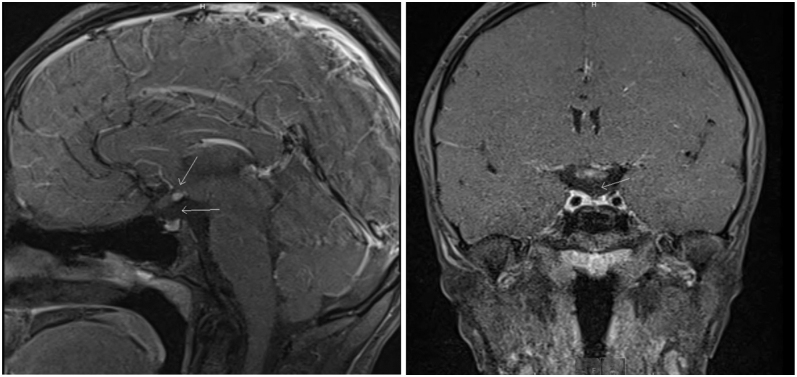
MRI demonstrating pituitary stalk interruption syndrome (PSIS). MRI demonstrating hypoplasia of the anterior pituitary with arrows denoting the absent infundibulum, findings compatible with PSIS.

Workup from the primary care physician confirmed a normal karyotype (46,XX). Her bone age was consistent with her chronological age, predicting an adult height of approximately 152.4 cm—notably lower than her calculated mid-parental height of around 167.6 cm (75th percentile). Even with arthrogryposis, her growth plates are able to be adequately visualized to assess her bone age accurately. Laboratory evaluation supported GH deficiency, hypothyroidism, and adrenal insufficiency (Table 1), and cortisol and GH stimulation testing showed subnormal responses (Table 2). An MRI (Fig. 1) performed three months later (June 2023) showed hypoplasia of the anterior pituitary, an absent infundibulum, and an ectopic, undescended posterior pituitary—findings compatible with pituitary stalk interruption syndrome (PSIS) in the setting of panhypopituitarism. Based on these findings, the patient was diagnosed with panhypopituitarism.

**Table 1:** Key hormone values during initial workup compared to 6-month follow-up after GH replacement.

Hormones	Initial workup	Follow-up*	Reference
TSH, μIU/mL	4.01	1.59	0.40–4.30
Free T4, ng/dL	0.56	0.75	0.70–1.37
IGF-1, ng/mL	12	365	133–416
IGF-1 Z-score, SD	−4.6	−0.2	−2.0 to +2.0
IGFBP3, mg/L	1.4	N/A	2.7–8.9
LH, mIU/mL	0.12	<0.02	0.04–10.80
FSH, mIU/mL	0.99	0.12	0.87–9.16
Estradiol, pg/mL	<2	4	≤142
Cortisol, μg/dL	3.5	N/A	2.97–23.4

IGFBP3, IGF-binding protein 3.

* Six months after GH replacement.

**Table 2:** Cortisol and GH stimulation test results.

Hormones	Baseline	2 h	4 h	4.5 h	5 h	Reference
Cortisol, μg/dL	2.8	N/A	1.2	1.9	3.8	≥18
GH, ng/mL	<0.1	<0.1	<0.1	<0.1	<0.1	≥10

GH, growth hormone.

## Treatment

Treatment began with hydrocortisone (9 mg/m^2^/day) and levothyroxine (37.5 μg daily). Repeat labs in July 2023 showed normalized thyroid hormone levels. At that time, GH replacement therapy had not yet been started due to concerns that it might exacerbate her hand contractures secondary to arthrogryposis. However, after extensive discussion of risks and benefits, the family indicated a preference to begin GH supplementation. Given the patient’s needle phobia, she was started on weekly subcutaneous Skytrofa injections (7.6 mg SQ weekly, 0.24 mg/kg/week) administered in the legs. The first dose was given in August 2023.

## Outcome and follow-up

**Figure 2:**
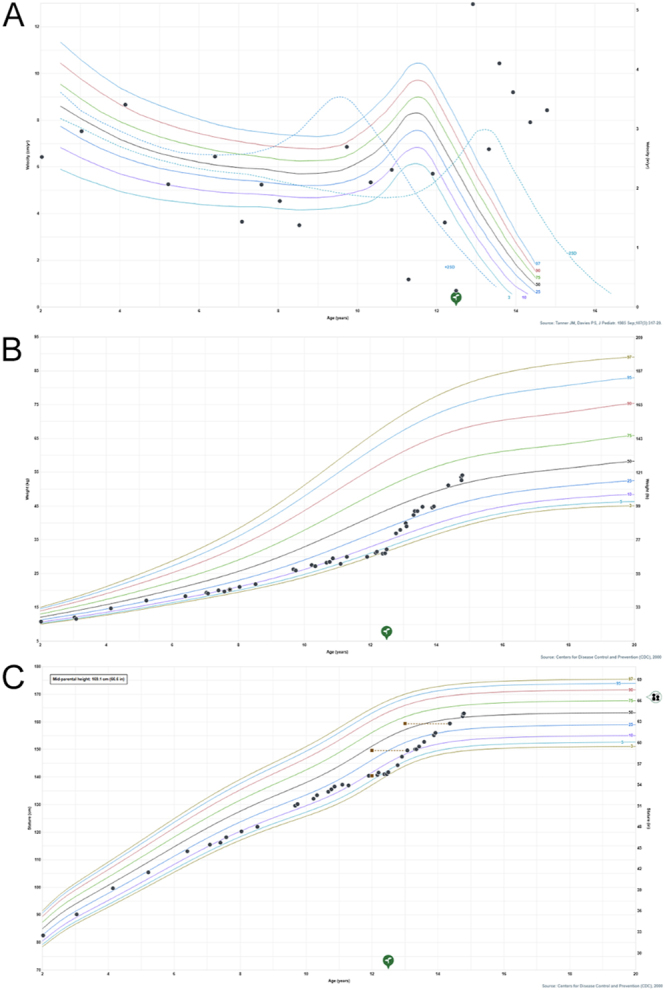
Growth chart and height-velocity chart peri-GH replacement therapy. Velocity growth chart (A): after initiation of GH replacement therapy, height improved from below 5th percentile to above 50th percentile. Chart (B): after initiation of GH replacement therapy, growth velocity increased from below 3rd percentile to above 97th percentile; green tree: time of GH therapy initiation. Parental sign: mid-parental (target height).

**Figure 3:**
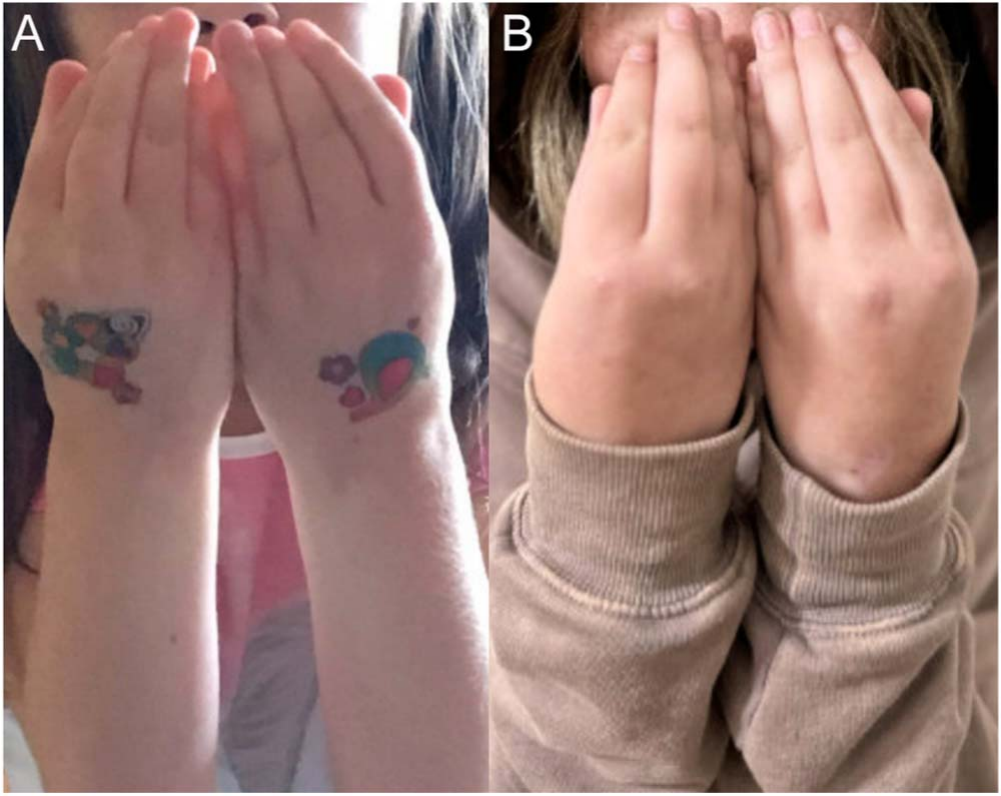
No change in distal arthrogryposis contractures before and after GH therapy. (A): before GH therapy; (B): over one year after GH therapy initiation.

At her three-month follow-up visit in November 2023, the patient had been adherent to the Skytrofa regimen and had not experienced adverse effects. Her growth velocity had increased to 8.7 cm/year (Fig. 2), and there was some evidence of breast development. By the next three-month follow-up visit in February 2024, she continued to adhere to her medications and reported outgrowing her pants, with a growth velocity recorded at 17.4 cm/year. A repeat bone age X-ray taken in February 2024 was consistent with her chronological age, and her predicted adult height had increased to 162.6 cm at this time, compared to the previous prediction of 152.4 cm. The family also noted subjective signs of puberty with beginning of breast development. At her most recent visit in June 2024, the patient had gone up a clothing size (now wearing size 14). Of note, despite the growth treatment, the patient and her mother deny any changes in her hand contractures (Fig. 3), which were supported by serial in-office examinations during their pediatric endocrinology visits. A longitudinal follow-up from orthopedic surgery was not indicated because her contractures did not warrant surgical intervention. On examination, she was found to be at Tanner stage 2 for breast development.

Since beginning Skytrofa, her height percentile had increased to above the 50th percentile (162.6 cm) at her latest follow up in Nov 2025 with her bone age in June 2025 of 13 years compared to a chronological age of 14 years and 4 months. The patient and her mother deny any worsening contractures, which is supported by an in-office examination. She is currently closely followed up in an endocrine clinic with weekly Skytrofa injections and continued treatment with daily levothyroxine and maintenance hydrocortisone.

## Discussion

This case highlights a rare co-occurrence of late-onset panhypopituitarism and distal arthrogryposis in a 12-year-old female. The patient presented with a significant growth delay, falling below the 5th percentile in height by age 12 despite a mid-parental height projecting to the 75th percentile. Laboratory evaluations confirmed deficiencies in GH, TSH, and ACTH. MRI revealed hypoplasia of the anterior pituitary, an absent infundibulum, and an ectopic posterior pituitary, findings consistent with PSIS. Initiation of GH therapy using weekly Skytrofa led to a significant improvement in growth velocity without aggravating her arthrogryposis-related joint contractures.

Late-onset panhypopituitarism is uncommon, with most cases diagnosed in infancy or early childhood when developmental delays are more apparent. Typical causes of late-onset panhypopituitarism include brain trauma, tumors, and structural abnormalities, such as PSIS. In this patient, the MRI findings suggest a congenital structural abnormality rather than an acquired cause; thus, her late presentation is atypical. This may be associated with progressive worsening of endocrine function in patients with PSIS and echoes the importance of consistent follow-up in this patient population (5).

Distal arthrogryposis is a subset of arthrogryposis multiplex congenita characterized by congenital contractures of distal joints, primarily affecting the hands and feet, with an estimated prevalence of ∼1 in 3,000 (6). Multiple types are classified by clinical features and genetic findings; the most likely phenotype here is distal arthrogryposis type 1, which involves hand contractures without significant craniofacial involvement (7). Gene associations reported for certain subtypes include *TNNT3*, *TNNI2*, *TPM2*, and *MYH3* (8).

To our knowledge, detailed reports on simultaneous co-occurrence of distal arthrogryposis and congenital panhypopituitarism are currently lacking. One possible explanation could be an underlying genetic cause affecting both musculoskeletal and endocrine development. Mutations in *MAGEL2* and *L1CAM* have been associated with congenital hypopituitarism and arthrogryposis in a small series, highlighting the need for further study (9). Genetic testing was not conducted in this case given the late onset of diagnosis of panhypopituitarism and successful treatment but will be considered.

Initiating GH therapy in patients with arthrogryposis presents specific challenges, centered on concern for potential exacerbation of joint contractures and GH’s effects on collagen synthesis (5). A single case report has described GH use in arthrogryposis multiplex congenita with multiple pituitary hormone deficiencies, but no follow-up data were provided. Accordingly, there is a theoretical risk that GH could worsen existing abnormalities or lead to new contractures, and clinicians may hesitate to initiate therapy. In this case, these concerns were addressed through shared decision-making. Although GH was initially deferred, the family elected to proceed to address a significant growth delay. The patient’s growth velocity increased to 17.4 cm/year at six months without any worsening of hand contractures.

Despite the theoretical risk of GH therapy worsening contractures, data from the National Cooperative Growth Study indicate that only a small proportion (0.1%) of patients with idiopathic short stature receiving GH reported arthralgia or arthritis (10). Mechanistically, distal arthrogryposis is a genetic condition that does not involve the GH axis; therefore, GH would not be expected to exacerbate this congenital condition. Nonetheless, this is a single case, and generalizations should be made with caution. We emphasize the importance of shared decision making and frequent follow-up in the use of GH therapy in patients with congenital contractures and the importance of further research in assessing the long-term outcomes of GH therapy in patients with coexisting panhypopituitarism and arthrogryposis.

## Declaration of interest

The authors declare that there is no conflict of interest that could be perceived as prejudicing the impartiality of the research reported.

## Funding

This research did not receive any specific grant from any funding agency in the public, commercial, or not-for-profit sector.

## Patient consent

Written informed consent for publication of their clinical details and clinical images was obtained from the parents of the patient.

## Author contribution statement

LM and GA contributed to data collection, literature review, manuscript drafting, and preparation of figures and tables. MP was involved in all aspects of case, from case conception to revision.
